# Cartographie de l’accès aux services de prise en charge de l’hypertension par les médecins généralistes au Burkina Faso en 2020

**DOI:** 10.11604/pamj.2024.48.173.34706

**Published:** 2024-08-13

**Authors:** Relwendé Aristide Yameogo, Dakaboue Germain Mandi, Joel Bamouni, Patrice Zabsonre, Nicolas Meda

**Affiliations:** 1 Département de Santé Publique, Unité de Formation et de Recherche des Sciences de la Santé, Université Joseph Ki-Zerbo, Ouagadougou, Burkina Faso,; 2 Service d'Informatique, CHU Tengandogo, Ouagadougou, Burkina Faso,; 3 Service de Cardiologie, CHU Yalgado Ouédraogo, Ouagadougou, Burkina Faso,; 4 Service de Médecine, CHU de Ouahigouya, Ouahigouya, Burkina Faso,; 5 Faculté de Médecine, Centre Universitaire Polytechnique de Ouahigouya, Ouahigouya, Burkina Faso,; 6 Département de Médecine, Unité de Formation et de Recherche des Sciences de la Santé, Université Joseph Ki-Zerbo, Ouagadougou, Burkina Faso

**Keywords:** Hypertension artérielle, réseau de soins, formation, cartographie, Afrique, Arterial hypertension, care network, training, mapping, Africa

## Abstract

**Introduction:**

l'hypertension artérielle (HTA) constitue un problème de santé publique au Burkina Faso. Sa prise en charge n'est pas du seul ressort du spécialiste mais nécessite la participation de tous les acteurs dans le cadre d'un réseau de soins avec une implication de ceux qui sont au premier plan. L'objectif de cette étude était d'analyser la capacité de la prise en charge de l'HTA au Burkina Faso et d'en fournir une cartographie.

**Méthodes:**

il s'est agi d'une enquête transversale en ligne auprès des médecins généralistes du Burkina Faso à travers les réseaux sociaux. L'échantillonnage a été faite sur la base du volontariat.

**Résultats:**

notre étude a concerné 182 médecins généralistes avec un sex-ratio de 2.7: 1. L'âge moyen des médecins était de 31 ans avec une expérience professionnelle moyenne de 2,7 ans. Le bilan minimum de l'OMS pour la prise en charge de l'HTA était disponible pour 80% des médecins et dans 74% des villes. La majeure partie des médecins (96%) se limitait à la bithérapie anti-hypertensive avec une prescription fréquente des inhibiteurs calciques (75,8%) des inhibiteurs de l'enzyme de conversion (51,6%) et des diurétiques (40,7%). L'avis des spécialistes était demandé en cas de non contrôle de l'HTA (52,8%) avec une faible interaction avec les médecins spécialistes: seulement 20,3% de contre-référence. Les médecins (93%) souhaitaient participer à un réseau de soins de prise en charge de l'HTA mais ont besoin de formation dans 98,4% des cas.

**Conclusion:**

le Burkina Faso a une répartition inégale des moyens de prise en charge de l'HTA. Les compétences des médecins doivent être renforcées pour améliorer la qualité de la prise en charge. Un meilleur management des ressources et la mise en place d'un réseau de soins permettraient de mieux coordonner les activités et améliorer la prise en charge de l'HTA.

## Abstract (en)

**Introduction:**

arterial hypertension (AH) is a public health problem in Burkina Faso. Its management is not the sole responsibility of the specialist but requires the participation of all healthcare actors within a care network, with the involvement of those at the forefront. This study aimed to analyse and map the capacity for managing hypertension in Burkina Faso.

**Methods:**

we conducted a cross-sectional online survey among general practitioners in Burkina Faso via social networks. Sampling was carried out voluntarily.

**Results:**

our study involved 182 general practitioners with a sex ratio of 2.7: 1. The average age of the physicians was 31 years with an average professional experience of 2.7 years. The WHO minimum assessment for the management of hypertension was available for 80% of doctors and in 74% of the towns. Most physicians (96%) limited their treatment to antihypertensive dual therapy, frequently prescribing calcium channel blockers (75.8%), converting enzyme inhibitors (51.6%), and diuretics (40.7%). Specialist advice was sought in the event of uncontrolled hypertension (52.8%), with little interaction with specialists: only 20.3% of counter-referrals. Most physicians (93%) wished to participate in an AH management care network, but 98.4% needed training.

**Conclusion:**

Burkina Faso has an uneven distribution of AH management resources. Physicians’ skills need to be enhanced to improve the quality of care. Better management of resources and establishing a care network would allow better coordination of activities and improve the management of hypertension.

## Introduction

L'hypertension artérielle (HTA) est l'un des principaux facteurs de risque de mortalité et de morbidité cardiovasculaires dans le monde [1,2]. C'est un facteur de risque cardiovasculaire majeur dans la survenue d'accidents vasculaires cérébraux, d'insuffisance cardiaque, d'insuffisance rénale et de maladies coronaires qui représentent les principales causes de décès dans le monde [3,4]. Au Burkina Faso, la prévalence de l'HTA était de 24.8% en 2015 selon l'enquête STEPS [5]. Dans une étude réalisée par Yaméogo NV en milieu hospitalier [6], les facteurs de risque cardiovasculaire (RCV) modifiables outre l'HTA étaient dominés par les dyslipidémies (29.8%) et le diabète (24.6%). Le risque cardiovasculaire global calculé selon la méthode de Framingham était élevé dans 24,8% et très élevé dans 19.9% des cas. Les patients ayant un RCV élevé à très élevé avaient un mauvais contrôle tensionnel (65%) et les patients qui avait des effets secondaires médicamenteux avaient un mauvais contrôle tensionnel dans 71% des cas. Malgré une large diffusion des recommandations scientifiques sous forme textuelle, on constate une certaine inefficacité de ces recommandations à modifier les comportements des médecins et la persistance de pratiques médicales variables avec un taux d'inertie thérapeutique très important (score inertie de 87% dans le service de cardiologie du CHU Yalgado Ouédraogo [7]. Les médecins généralistes, premiers contacts avec les patients, sont repartis dans les différents districts sanitaires du pays et ne disposent pas d'un guide de prise en charge de l'HTA [8]. Nous faisons l'hypothèse que les ressources de prise en charge de l'HTA sont reparties de manière inégale sur le territoire. L'objectif de notre étude est d'analyser la capacité de prise en charge de l'HTA par les médecins généralistes au Burkina Faso et d'en fournir une cartographie.

## Méthodes

### Période et type d'étude

Nous avons réalisé une enquête transversale en ligne à partir de l'outil Google Forms® du 01^er^ novembre 2019 au 20 février 2020 au Burkina Faso. Cette enquête a concerné les médecins généralistes pratiquant au Burkina Faso quel que soit la structure d'exercice publique ou privée. Le formulaire a été envoyé dans les groupes de réseaux sociaux des médecins Burkinabé (Facebook®, Telegram® et WhatsApp®). Le nombre de médecins contactés à travers ses outils était de 2243. L'échantillonnage était basé sur la volonté du médecin à participer à l'enquête.

### Cadre de l'étude

**Figure 1:**
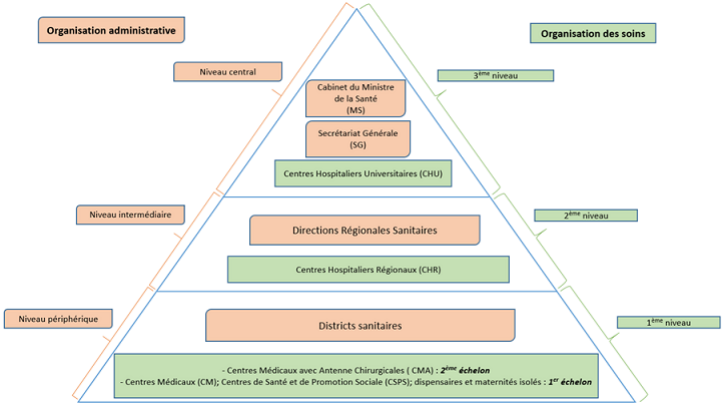
organisation administrative et de l'offre de soins selon la pyramide sanitaire

Le Burkina Faso est subdivisé en 13 régions, 45 provinces, 350 départements, 351 communes (49 communes urbaines et 302 communes rurales) et 8 228 villages [9]. Les structures publiques de soins sont organisées en trois niveaux qui assurent des soins primaires, secondaires et tertiaires Figure 1 [10]: le premier niveau comprend deux échelons: le premier échelon de soins est composé des centres médicaux, des centres de santé et de promotion sociale (CSPS), des centres médicaux, des dispensaires et maternités isolés. Le deuxième échelon sert de référence pour le premier échelon. Il est représenté par les centres médicaux avec antenne chirurgicale (CMA); le deuxième niveau est représenté par les centres hospitaliers régionaux (CHR), ils servent de référence pour les CMA; le troisième niveau est constitué par les centres hospitaliers universitaires et constituent le niveau de référence le plus élevé. Ils servent également de cadre de formation pour les différentes catégories de personnels et de cadre de recherches.

### Variables et collecte des données

Les données ont été collectées directement auprès des médecins ayant participé à l'étude. Les données collectées concernaient: les caractéristiques sociodémographiques des participants: âge, sexe, lieu de pratique médicale, expérience professionnelle; la prescription des bilans pour la prise en charge de l'HTA: hémogramme, glycémie, créatininémie, protéinurie de 24 heures, acide urique, bilan lipidique (cholestérol total, LDL, triglycérides), électrocardiogramme, échocardiographie cardiaque, fond d'œil; la disponibilité des bilans dans leur ville d'exercice dans le privé et/ou le public; les molécules anti hypertenseurs régulièrement prescrites et le nombre d'antihypertenseur; la période de demande de l'avis du médecin spécialiste; les besoins de formation en matière d'HTA: physiopathologie, diagnostic, évaluation du risque cardiovasculaire, prescription des anti hypertenseurs, gestion des complications, suivi des patients hypertendus, gestion des comorbidités Le bilan minimum de l’Organisation mondiale de la Santé (l'OMS) était constitué de l'hémogramme, la glycémie, la créatinine, le bilan lipidique (cholestérol total, GDL, LDL, Triglycérides), l'ionogramme sanguin, les protéinuries de 24 heures et l'électrocardiogramme. Le bilan complet était constitué de bilan minimum de l'OMS associé à l'échocardiographie Doppler et du fond œil si disponible. Les données concernant la répartition des cardiologues sur le territoire national ont été recueillies auprès de la Société de Cardiologie du Burkina (SOCARB).

### Taille de l'échantillon

**Figure 2:**
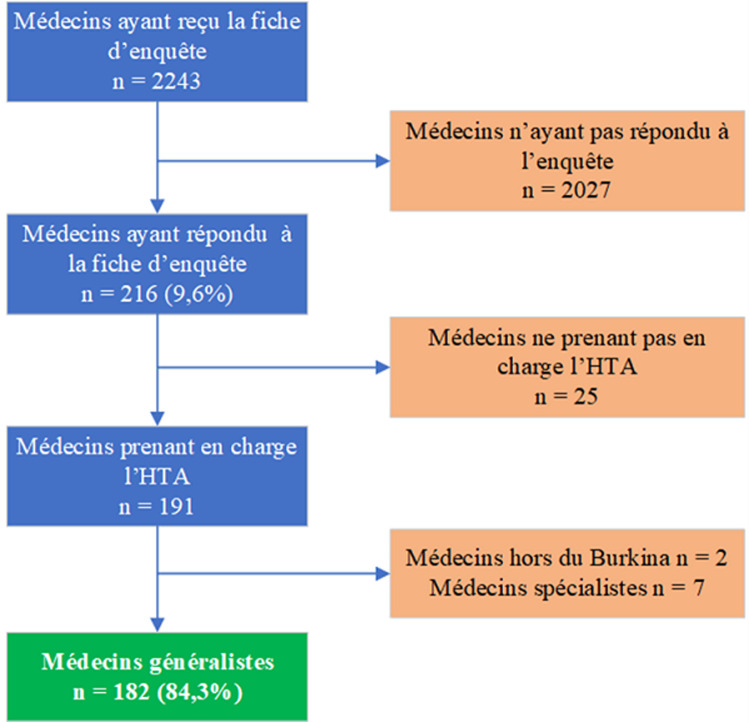
sélection de la population d'étude

Les nombres de médecins ayant participé à l'étude était de 216 soit un taux de réponse de 9,6%. La participation était basée sur le volontariat et la motivation des médecins et peut entrainer un biais de représentativité de certaines villes. La Figure 2 montre le processus de sélection des médecins enquêtés. Les médecins spécialistes de l'hypertension artérielle, les médecins résidant hors du Burkina Faso et les médecins ne prenant pas en charge l'hypertension artérielle ont été exclus de notre étude.

### Analyse des données

Les données ont été analysées avec le logiciel RStudio 2021.09.1+372. Les données qualitatives ont été calculées sous forme de pourcentage et les données qualitatives sous forme de moyenne ± écart type. Les cartes ont été réalisées à partir des données de géolocalisation des différentes villes où travaillaient les médecins et les données sur la disponibilité des examens complémentaires. Le logiciel QGIS a été utilisé à cet effet.

### Considérations éthiques

Nous avons reçu l'autorisation de la Commission Informatique et Libertés (CIL) pour la collecte des données en ligne. Les données concernant les identités, les emails et les adresses IP des participants n'étaient pas collectés pour le respect de l'anonymat et de la confidentialité des données.

## Résultats

### Population d'étude

Le formulaire a été envoyé à 2243 médecins avec un retour de réponse de 216 médecins soit un taux de réponse de 9,6% dont 25 médecins ont été exclus car ne prenant pas en charge l'hypertension artérielle et deux autres médecins non résidant au Burkina Faso. Notre étude a concerné 182 médecins généralistes intervenant dans la prise en charge de l'hypertension artérielle. La Figure 2 montre le processus de mise en place de l'échantillon de notre étude. Les médecins inclus dans cette étude provenaient de 53 villes du Burkina Faso.

### Caractéristiques sociodémographiques de la population

L'âge moyen de la population était de 31 ± 2,4 ans avec un âge minimum de 26 ans et maximum de 41 ans avec un sex-ratio de 2,7. Le Centre Médical avec Antenne chirurgicale (CMA) comme lieu d'exercice était retrouvé dans 33,5% des cas (n = 61). Les médecins avaient une expérience professionnelle moyenne de 2,7 ± 1,4 ans avec un minimum de zero anet un maximum de 8 ans. Le Tableau 1 montre la répartition des caractéristiques sociodémographiques des enquêtés.

**Tableau 1:** caractéristiques sociodémographiques des médecins généralistes

Variables	Fréquence	Pourcentage
**Sexe**		
Masculin	133	73,1
Féminin	49	26,9
**Age moyen**	31 ± 2,4 ans [26 – 41]	
**Groupe d’âge (année)**		
[25 – 30]	91	50,0
[30 – 35]	80	44,0
[35 – 40]	10	05,5
>40	01	00,5
**Lieu d’exercice**		
Structure publique		
CHR	33	18,1
CHU	19	10,4
CM	37	20,3
CMA	61	33,5
Structure privée	32	17,6
**Expérience professionnelle**		
[0 – 2]	97	53,3
[2 – 4]	68	37,4
[4 – 6]	11	06,0
>6 ans	06	03,3

CHR: Centre Hospitalier Régional; CHU: Centre Hospitalier Universitaire; CM: Centre Médical; CMA: Centre Médical avec Antenne chirurgicale

### Prescription des bilans lors de la prise en charge de l'HTA

**Figure 3:**
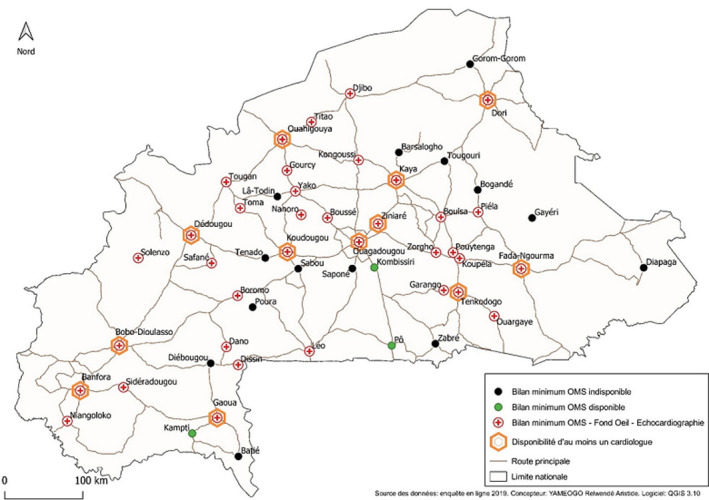
cartographie de la disponibilité des bilans complémentaires et des cardiologues

Sur les 182 médecins, 49,5% (n = 90) et 38,5% (n = 70) prescrivaient respectivement une créatinine et un électrocardiogramme au moment du diagnostic. La créatinine et l'électrocardiogramme étaient prescrits respectivement dans 62,7% (n = 114) et 50% (n = 91) des cas au moins une fois dans l'année. Le Tableau 2 montre la répartition des prescriptions de bilan complémentaires par les médecins généralistes. Les bilans complémentaires les plus prescrits au moment du diagnostic étaient la glycémie, la créatininémie et le bilan lipidique avec 49,4%, 48,4% et 43,4% des cas respectivement (Tableau 3). Le bilan minimum de l'OMS pour la prise en charge de l'HTA était réalisable par 80% (n = 145) des médecins et dans 73,6% (n = 39) des villes. Le bilan complet était réalisable dans 67,9% (n = 36) des villes et accessible à 72,5% (n = 132) des médecins. La Figure 3 et le Tableau 3 montrent la disponibilité des bilans complémentaires dans les structures de soins.

**Tableau 2:** prescription des bilans complémentaires par les médecins généralistes

	Au moment du diagnostic		En fonction du besoin		Tous les six mois		Une fois par an		Pas du tout	
	n	%	n	%	n	%	n	%	n	%
Hémogramme	29	15,9	07	03,8	31	17,0	29	15,9	13	07,1
Glycémie	90	49,4	42	23,1	71	39,0	43	23,6	02	01,1
Créatinine	88	48,4	41	22,5	77	42,3	42	23,1	01	00,5
Ionogramme sanguin	77	42,3	60	33,0	63	34,6	27	14,8	03	01,6
Bilan lipidique	79	43,4	32	17,6	51	28,0	54	29,7	04	02,2
Acide urique	58	31,9	46	25,3	42	23,1	41	22,5	20	11,0
Protéinurie de 24 heures	44	24,2	62	34,1	24	13,2	45	24,7	22	12,1
Fond œil	66	36,3	43	23,6	17	09,3	74	40,6	17	09,3
Électrocardiogramme	70	38,5	38	20,9	31	17,0	60	33,0	07	03,8
Echocardiographie Doppler	55	30,2	74	40,6	15	08,2	48	26,4	18	09,9

**Tableau 3:** disponibilité des examens dans les structures de soins auprès des médecins

	Publique		Privée		Associative		Non disponible	
	n	%	n	%	N	%	n	%
Hémogramme	168	92,3	55	30,2	11	06,0	04	02,2
Glycémie	166	91,2	56	30,8	10	05,5	02	01,1
Créatinine	166	91,2	57	31,3	11	06,0	03	01,6
Ionogramme sanguin	122	67,0	76	41,8	10	05,5	14	07,7
Bilan lipidique	102	56,0	87	47,8	10	05,5	29	15,9
Acide urique	139	76,4	69	37,9	11	06,0	10	05,5
Protéinurie de 24 heures	112	61,5	87	47,8	08	04,4	26	14,3
Fond œil	88	48,4	74	40,6	07	03,8	46	25,3
Electrocardiogramme	108	59,3	78	42,8	02	01,1	31	17,0
Echocardiographie Doppler	84	46,2	92	50,5	05	02,7	35	19,2

### Prescription médicamenteuse des médecins généralistes

Les molécules les plus fréquemment prescrits par les médecins généralistes étaient les inhibiteurs calciques, les inhibiteurs de l'enzyme de conversion de l'angiotensine II et les diurétiques (Tableau 4). L'utilisation de la bithérapie était notée chez 96,2% (n = 175) des médecins. Le Tableau 5 montre la répartition des associations du nombre d'anti hypertenseurs faites par les médecins.

**Tableau 4:** molécules prescrites par les médecins généralistes

	Plus souvent		Parfois		Rarement		Pas du tout	
	n	%	n	%	n	%	n	%
Béta bloquant	03	01,6	69	37,9	45	24,7	65	35,7
Inhibiteurs calciques	138	75,8	39	21,4	01	00,5	04	02,2
IEC	94	51,6	66	36,3	15	08,2	07	03,8
ARA II	04	02,2	38	20,9	53	29,1	87	47,8
Diurétiques	74	40,6	83	45,6	13	07,1	12	06,6
Anti-hypertenseurs centraux	17	09,3	61	33,5	54	29,7	49	26,9

**Tableau 5:** association des molécules anti hypertenseurs par les médecins

	Fréquence	Pourcentage
Bithérapie	175	96,2
Trithérapie	37	20,3
Quadrithérapie	01	00,5
Quintuple thérapie	01	00,5

### Collaboration des médecins généralistes avec les spécialistes

les médecins pensaient dans 99% (n = 180) des cas que la prise en charge de l'HTA n'est pas du seul ressort du spécialiste et l'avis du spécialiste était demandé dans 52,8% (n = 96) des cas lorsque l'HTA n'était pas contrôlée. Le Tableau 6 montre la répartition en fonction du moment de demande de l'avis du spécialiste. Les contre-références étaient réalisées dans 20,3% (n = 37) des cas.

**Tableau 6:** répartition du moment de demande de l'avis du spécialiste

	Fréquence	Pourcentage
HTA non contrôlée	96	52,8
Dès le diagnostic	21	11,5
Lors des complications	57	31,3
Systématiquement	8	04,4
Total	182	100,0

### Participation à un réseau de soins

Les médecins aimeraient participer à un réseau de soins de prise en charge de l'HTA dans 93% (n = 169) et 90% (n = 163) se disaient prêt à participer à une prise en charge sur le long terme de l'HTA. Un besoin de formation complémentaire a été demandé par 98,4% (n= 179) médecins. Les thématiques sont présentées dans le Tableau 7.

**Tableau 7:** répartition des besoins de formation complémentaire

	Fréquence	Pourcentage
Physiopathologie	57	31,3
Diagnostic	32	17,6
Évaluation du risque cardiovasculaire	101	55,5
Prescription des anti hypertenseurs	149	81,9
Gestion des complications	151	83,0
Suivi des patients hypertendus	144	79,1
Gestion des comorbidités	154	84,6

## Discussion

L'implication des médecins généralistes est très importante dans la prise en charge des patients hypertendus et ils ont la volonté d'y contribuer; 90% des médecins souhaiteraient participer à la prise en charge à long terme de l'HTA. Il s'agit d'une pathologie qui touche aussi bien le milieu rural que le milieu urbain [5]. En effet, l'HTA est la première cause de mortalité dans les hôpitaux du Burkina Faso [11,12] et septième cause de décès dans les formations sanitaires de base [13]. Or, il existe peu de médecins spécialistes pour un bon maillage territorial. En plus, même si la capacité des villes (74% des villes sont capables de réaliser le bilan minimum de l'OMS) à prendre en charge l'HTA est satisfaisante, on constate une répartition inégale des ressources sur le territoire. La mise en place d'un réseau numérique de soins permet non seulement d'uniformiser la prise en charge de l'HTA sur une grande échelle en fonction des recommandations; mais permet également de mieux gérer la mobilité des agents de santé et des patients à travers un accès unique pour les agents et un dossier unique pour les patients. En effet, dans notre travail, la majorité de nos médecins ont une expérience professionnelle de moins quatre ans (90,7%), témoin de la forte mobilité des médecins dans le cadre de la spécialisation et les cardiologues sont localisés au niveau des chefs-lieux des régions [14] avec une forte concentration dans les villes de Bobo Dioulasso et de Ouagadougou; la télémédecine permettrait une meilleure exploitation des ressources disponibles dans les villes où ils n'existent pas de cardiologues et une meilleure interaction entre le cardiologue et le médecin généraliste à travers un dossier communicant de l'HTA. Le cardiologue se déplacerait uniquement pour des cas sélectionnés en collaboration avec le médecin généraliste. Ainsi, le réseau de soins va permettre une mutualisation efficiente des ressources humaines et matérielles au sein du ministère de la santé et un ancrage territorial des soins de santé.

Actuellement, les cardiologues sont obligés de programmer des déplacements dans les différentes localités, cela entraine un accès inégal au soins cardiologiques car les déplacements se font vers les zones les plus « rentables » (où la masse critique de patients est importante). Le nouveau code de santé publique du Burkina Faso, en cours de validation depuis juin 2020, reconnait la santé numérique comme pratique médicale. Ils restent encore à mettre en place les décrets d'applications sur le terrain et proposer un modèle économique viable surtout dans un contexte de gratuité et de subvention importantes des soins. L'avènement de la maladie à COVID-19 a montré les limites de notre système de santé actuel [15,16]; durant cette période, les patients hypertendus étaient considérés à risque de développer des formes graves de la maladie à COVID-19 et l'aménagement des structures de soins pour mieux accueillir les patients dans les CHU et CHR qui sont à la fois les lieux de pratiques des cardiologues à entrainer une réduction de l'offre de soins pour ces patients (certains hôpitaux ont été réquisitionnés pour la prise en charge de la maladie à COVID-19) [17]. Il est plus qu'important de mettre en place des systèmes de santé résilients et durables pour une meilleure prise en compte des besoins des communautés. Le numérique peut constituer ce levier et mettre en place un pont entre la pratique médicale clinique et la santé publique [18]; mais cela nécessite de prendre en compte les enjeux éthiques (protection des données, inclusion des minorités, confidentialité des données, accès aux outils informatiques et à internet) et le respect des règles de la pratique médicale.

La mise en place de ce réseau va nécessiter l'élaboration et la mise à disposition d'un guide de prise en charge de l'HTA adapté à notre contexte. La formation des médecins s'avère importante afin que tous les acteurs soient au même niveau d'information. À cet effet, un diplôme universitaire de prise en charge de l'HTA est en cours d'ouverture à l'université Joseph Ki-Zerbo de Ouagadougou et devra servir de support pour une meilleure organisation de la prise en charge de l'HTA au Burkina Faso à l'image de pathologies comme le VIH, le paludisme ou la tuberculose. Mais, il faudra une implication des autorités sanitaires et universitaires pour rendre cette formation accessible à un plus grand nombre de médecins afin de faciliter la constitution d'un réseau de santé bien construit et solide. Le mode d'échantillonnage de notre population peut entrainer des biais de sélection, à savoir ceux qui étaient motivés pour participer à l'étude. Cette technique de collecte limitait également la participation des médecins n'ayant pas de compétences numériques ou d'accès à internet. Mais le regroupement des médecins en fonction des villes de résidence permettaient de palier les insuffisances de nombres de participants et mettre en évidence la disponibilité des infrastructures pour la prise en charge des patients hypertendus dans les villes.

## Conclusion

Notre étude montre une répartition inégale des moyens de prise en charge de l'HTA au Burkina Faso. Elle montre par ailleurs une volonté des médecins généralistes à s'impliquer dans la prise en charge de l'HTA; mais cela nécessite un renforcement des compétences de ces derniers sur la prise en charge de l'HTA. Le projet d'ouverture d'un diplôme universitaire d'hypertension artérielle constitue une belle opportunité; mais il faudra une implication des autorités sanitaires pour la rendre accessible à l'ensemble des acteurs de la prise en charge. Un meilleur management des ressources et la mise en place des réseaux de soins numérique permettraient une amélioration considérable de la prise en charge de cette pathologie au Burkina Faso.

### Etat des connaissances sur le sujet

- *L'absence de recommandations de bonne pratique pour la prise en charge de l'hypertension artérielle;*
- *Faible implication des médecins généralistes dans la prise en charge de l'hypertension artérielle.*

### Contribution de notre étude à la connaissance

- *Une répartition inégale des ressources de prise en charge de l'HTA;*
- *Une volonté des médecins généralistes à la participation de la prise en charge de l'HTA;*
- *Une nécessité de mise en place d'une formation continue pour les médecins généralistes.*
